# COVID-19 in Pediatric Intensive Care Units in Poland, PAPITCO-19 Study (Polish Analysis of PICU Trends during COVID-19)

**DOI:** 10.3390/jcm12123983

**Published:** 2023-06-12

**Authors:** Maria Damps, Elżbieta Byrska-Maciejasz, Małgorzata Kowalska, Jowita Rosada-Kurasińska, Beata Rybojad, Joanna Sordyl, Marzena Zielińska, Alicja Bartkowska-Śniatkowska, on behalf of the PAPITCO-19 Group

**Affiliations:** 1 Department of Anaesthesiology and Intensive Care, Upper Silesian Child Health Centre, Faculty of Medical Sciences in Katowice, Medical University of Silesia, 40-752 Katowice, Poland; 2 Department of Anaesthesiology and Intensive Therapy, University Children’s Hospital of Krakow, 30-663 Krakow, Poland; a.e.maciejasz@wp.pl; 3 Department of Epidemiology, School of Medicine in Katowice, Medical University of Silesia, 40-514 Katowice, Poland; mkowalska@sum.edu.pl; 4 Department of Paediatric Anaesthesiology and Intensive Care, Poznań University of Medical Sciences, 60-806 Poznan, Poland; jrosada@ump.pl (J.R.-K.); asniatko@ump.edu.pl (A.B.-Ś.); 5 Department of Anaesthesiology and Intensive Therapy, Faculty of Medicine, Medical University of Lublin; 20-035 Lublin, Poland; beata.rybojad@umlub.pl; 6 Department of Oncology, Hematology, and Chemotherapy, Upper Silesian Child Health Centre, 40-752 Katowice, Poland; joanna_sordyl@wp.pl; 7 Department of Anesthesiology and Intensive Care, Wrocław Medical University, 50-556 Wrocław, Poland; marzena.zielinska@gmail.com; 8 Department of Paediatric Anesthesiology and Intensive Care, University Clinical Hospital in Wrocław, 50-556 Wrocław, Poland

**Keywords:** SARS-CoV-2, pandemic, children infection, respiratory failure

## Abstract

Background: Children suffering from COVID-19 constitute about 10% of the entire population infected with the virus. In most of them, we observe asymptomatic or mild courses; however, about 1% of affected children require a stay in a paediatric intensive care unit (PICU) due to the course of the disease becoming severely life-threatening. The risk of respiratory failure, as with adults, is associated with the coexistence of concomitant diseases. The aim of our study was to analyse patients admitted to PICUs due to the severe course of their SARS-CoV-2 infection. We studied epidemiological and laboratory parameters, as well as the endpoint (survival or death). Methods: A retrospective multi-centre study, the analysis covered all children with a confirmed diagnosis of SARS-CoV-2 virus infection who were admitted to PICUs in the period from November 2020 to August 2021. We studied epidemiological and laboratory parameters, as well as the endpoint (survival or death). Results: The study analysed 45 patients (0.075% of all children hospitalised in Poland due to COVID-19 at that time). Mortality calculated in the entire study group was 40% (*n* = 18). Statistically significant differences between the compared groups (survived and died) concerned the parameters of the respiratory system. Lung Injury Score and the Paediatric Sequential Organ Failure Assessment were used. A significant correlation between disease severity and the patient’s prognosis was shown by the liver function parameter AST (*p* = 0.028). During the analysis of patients requiring mechanical ventilation and assuming survival as the primary outcome, a significantly higher oxygen index on the first day of hospitalisation, lower pSOFA scores and lower AST levels (*p*: 0.007; 0.043; 0.020; 0.005; 0.039, respectively) were found. Conclusions: As with adults, children with comorbidities are most frequently at risk of severe SARS-CoV-2 infection. Increasing symptoms of respiratory failure, the need for mechanical ventilation and persistently high values of aspartate aminotransferase are indicators of poor prognosis.

## 1. Introduction

In 2020, which was the first year of the COVID-19 pandemic, the population of paediatric patients accounted for 9.6–13.6% of the total number of patients diagnosed with SARS-CoV-2 virus infection [1]. Of this group, less than 1% required intensive care [2]. As with adults, the risk of death was associated with comorbidities, the most important of which were neurological diseases related to immobilisation, chronic respiratory failure and heart defects [3]. As a result of the experience gained during the treatment of adults infected with SARS-CoV-2, the diagnosis and treatment of infected children also improved [1]. At the end of April 2020, a new disease entity called Paediatric Inflammatory Multisystem Syndrome, temporally associated with SARS-CoV-2 (PIMS), appeared [4]. Based on data from the US, it can be estimated that PIMS develops in about 1/1000 children infected with SARS-CoV-2 [5]. PIMS is an acute and potentially serious generalised inflammatory syndrome that can lead to complications. Unlike the severe course of COVID-19, PIMS usually affects children with an unremarkable medical history. Subsequent waves of the pandemic have increasingly involved the youngest patients [2]. By November 2021, as many as 17,000 children were hospitalised in Poland due to SARS-CoV-2 infection. Until then, we had recorded 91,000 deaths in adult patients and 29 deaths in children under the age of 14 [6]. As in adults, the SARS-CoV-2 virus mainly affects the respiratory system; the infection causes fever and/or coughing, and finally acute respiratory failure. In some patients, we observed a fulminant course of infection, leading in some cases to treatment failure. Most studies have analysed the entire population of children infected with the SARS-CoV-2 virus [1,2,3,7], so this provided us with the basis for conducting our multicentre study. The purpose of our research was to provide information for healthcare workers and improve the care of infected and severely ill paediatric patients. We also tried to find “red flag” markers predicting poor outcome.

## 2. Materials and Methods

### 2.1. Data Source

A retrospective multi-centre study was conducted in 10 leading clinical Paediatric Intensive Care Units (centres with the number of patients are summarised in Table 1). Our research only included children admitted to Paediatric Intensive Care Units (PICUs) with confirmed COVID-19 and in a life-threatening state. The data come from all the leading paediatric centres in the country and reflect the actual clinical and epidemiological situation, which in the authors’ opinion, proves the high practical usefulness of the study.

In each of the departments, one person was appointed to be responsible for entering demographic, epidemiological, and clinical data into a common EXCEL spreadsheet. The analysis covered all children with a confirmed diagnosis of SARS-CoV-2 virus infection who were admitted to PICUs, in accordance with the Polish admission criteria, in the period from November 2020 to August 2021.

### 2.2. Reporting and Outcome

We analysed 45 children admitted to PICUs with SARS-CoV-2 virus infection confirmed via PCR (without variant identification). The analysis covered demographic, epidemiological, and laboratory data, including the following information: patient referral to PICU—referral from home, Hospital Emergency Department (ED), or Paediatric/Infectious Diseases Department; time elapsed from finding a positive result of SARS-CoV-2 to admission to the ward; comorbidities; congenital defects; results of laboratory tests; Paediatric Sequential Organ Failure Assessment (pSOFA) Score, with the following variables: PaO_2_:FiO_2_, platelet count, bilirubin, mean arterial pressure by age group, or vasoactive infusion, Peadiatric Glasgow Come Scale, creatinine by age group; the lung injury score (LIS) based on four criteria, which were hypoxemia, respiratory compliance, chest radiographic findings, and the level of positive end-expiratory pressure [5] (each criterion receives a score from zero to four according to condition severity, then the scores are summed across all criteria: 0 points indicates no lung injury, less than 2.5 points indicates mild-to-moderate lung injury, more than 2.5 points indicates presence of severe ARDS); treatment applied; need to support the respiratory system; oxygen demand; oxygenation index—the quotient of the partial pressure of oxygen in the arterial blood (PaO_2_) and the oxygen content in the breathing mixture (FiO_2,_ presented as a decimal fraction); and the length of PICU stay. The collected data were analysed to identify the predictors of COVID-19 survival in this group of patients. In addition, we performed a comparative analysis of mechanically ventilated patients divided into the groups of those who survived or died.

The study was approved by the Institutional Review Board of Medical University of Silesia (KE No PCN/CBN/0052/KB/43/23) and conformed to the provisions of the 1995 Declaration of Helsinki (as revised in Edinburgh, 2000).

### 2.3. Statistical Analysis

Statistical analysis was performed using a standard software package (STATISTICA 13 software (STATSOFT; Statistica, Tulsa, OK, USA) and PQStat (PQStat Software (2022), PQStat v.1.8.4.162). For continuous variables, the mean values, standard deviations (SD), and median were estimated. For categorical variables, the absolute values (*n*) and relative values (%) were assessed. The Shapiro–Wilk test was used to evaluate the normality of the distribution of quantitative data. In the case of abnormal distributions, the Mann–Whitney U test was used to compare continuous variables. The stochastic independence chi-square test with Yates’s correction was used to compare categorical variables. The value of *p* ≤ 0.05 was considered to be statistically significant. The odds ratio (OR) with a 95% confidence interval (CI) was calculated for the occurrence of death in children treated with mechanical ventilation compared to oxygen therapy; in patients with two or more comorbidities compared to children with a maximum of one concomitant disease and in children with prolonged hospitalisation (>10 days) in the ICU compared to patients with a stay shorter than 10 days.

## 3. Results

The study analysed 45 patients (25 males and 20 females) treated in PICUs in the period from November 2020 to August 2021. This number is 0.075% of all children hospitalised in Poland due to COVID-19 at that time [6]. The median age of patients was 2 years (IQR 11). Patients < 2 years of age accounted for 44% of the respondents, and children > 9 years of age accounted for 28.8%. The compared groups (survived and died) did not differ significantly in terms of sex, age, BMI (Body Mass Index), period (days) from diagnosis of infection to admission to the Intensive Care Unit, or comorbidities. Most children were referred from the COVID-19 ward (*n* = 12; 26.6%), the Paediatric Department (*n* = 12; 26.6%), or directly from home (*n* = 9; 20%). Comorbidities were found in as many as 40 subjects (88.8%). The most common comorbidities were neurological disorders (*n* = 20), immobilisation (*n* = 13), and respiratory failure (*n* = 12). Nine patients had congenital heart diseases in the form of either a ventricular septal defect (*n* = five), patent ductus arteriosus (*n* = one), pulmonary regurgitation (*n* = one), left heart hypoplasia (*n* = one), or common arterial trunk (*n* = one).

The characteristics of the study groups are presented in Table 2. Of the 45 patients, only 7 (16%) did not require mechanical ventilation, while the remaining 38 (84%) children required intubation and mechanical ventilation, and mortality in this group was 44.7% (*n* = 17). One child was treated with ECMO but did not survive. Mortality calculated in the entire study group was 40% (*n* = 18). The most common cause of death was the occurrence of bacterial and fungal superinfections with the development of septic shock (*n* = 10), cerebral edema as a result of CNS thrombovascular complications (*n* = 5), and right heart failure (*n* = 3).

Statistically significant differences between the compared groups (survived and died) concerned the parameters evaluating the efficiency of the respiratory system, i.e., pO_2_ on admission, SatO_2_ (arterial blood saturation) on admission, FiO_2_ used on the first day, the oxygen index on the first and third day (Table 3), and LIS on the first day (*p*-values: 0.018, 0.018, 0.020, 0.003, 0.006, and 0.047, respectively). In addition, statistically significant differences in pSOFA (Paediatric Sequential Organ Failure Assessment) scores on the first, third, and fifth day of intensive therapy were found in the study groups (*p* = 0.012, 0.007, and 0.001, respectively Table 3). A significant correlation between disease severity and the patient’s prognosis was shown by the liver function parameter, AST (*p* = 0.028, Table 3). The other assessed parameters did not differ significantly (Table 3). The applied treatment is presented in Table 4. Most of the children received steroid therapy and anticoagulant treatment. Immunoglobulins, convalescent plasma, and remdesivir were used less frequently. Three patients received tocilizumab. The average number of days of treatment in the ICU was 16 for patients discharged, and 10 for those who died. When analysing patients requiring mechanical ventilation and assuming survival as the primary outcome, a significantly higher oxygen index on the first day of hospitalisation; lower pSOFA scores on the first, third, and fifth days; and lower AST levels (*p*: 0.007; 0.043; 0.020; 0.005; 0.039, respectively) were found. The time from positive test to ICU admission for mechanically ventilated patients who died was not statistically different from the time for ventilated survivors; *p* = 0.247 (for the dead: 5.24 days on average ± 10.59, median 1.0; for “survivors”: 1.63 ± 2.63, median 2.13).

The results are presented in Table 5. The risk of death was 13.5 times higher in the ventilated group but did not differ significantly (OR = 13.53; 95% CI 0.72 to 253.73; *p* = 0.081).

## 4. Discussion

We report the first Polish study of paediatric life-threating forms of novel COVID-19 disease in children with severe SARS-CoV-2 virus infection and symptoms of impending respiratory and/or multiple organ failure requiring treatment in Paediatric Intensive Care Units. The key role in the mechanism of infection is played by the spike (S) protein, which is present on the surface of the virus and has an affinity for the ACE2 receptor protein (agiotensin-converting enzyme 2). The fusion of these two proteins allows viral particles to enter host cells. ACE2 is a receptor found on the mucosa of the upper and lower respiratory tract; enterocytes of the small intestine; in the kidneys, heart, testes, and cholangiocytes; and in the endothelium of blood vessels [8]. There may be a cytokine storm and rapid release of inflammatory mediators, e.g., pro-inflammatory cytokines (TNF alpha, IL-1, IL-6), anti-inflammatory cytokines (e.g., interleukin 10 and interleukin 1 receptor antagonists), and numerous oxygen free radicals and coagulation factors. Cytokines signal and stimulate cells of the immune system, including macrophages and T lymphocytes, to migrate towards the site of infection. Such a strong response from the immune system is observed in about 5% of patients [8]. The consequence of these disorders is insufficient oxygenation of the blood and deteriorating respiratory function, which may lead to Acute Respiratory Distress Syndrome (ARDS) [7]. The consequence of hypoxia and the development of a systemic inflammatory reaction can be multiple organ failure and shock. The need to use invasive ventilation with high-pressure parameters may secondarily cause circulatory failure, which will make it even more difficult to maintain proper systemic homeostasis.

In our study, the proportion of intubated children (84%) was higher than that reported in a similar study by Garcia-Salido et al., where 61.1% of children required intubation [9], or by Shekerdemian et al., where only 38% of children required mechanical ventilation [10]. Of the 45 patients, only 7 (16%) did not require mechanical ventilation (of those, 5 were treated with high-flow oxygen therapy using Airwo, and the other 2 patients were treated with low-flow passive oxygen therapy via the nasal canula), while the remaining 38 (84%) patients required intubation and invasive mechanical ventilation using the BiPAP, ASV, PCV modes (37 patients from the 1st day of admission, and 1 patient after the unsatisfactory effect of high-flow oxygen therapy required intubation and invasive ventilation from the 2nd day of hospitalisation in the ICU). Oxygen therapy is beneficial for patients with hypoxia to keep oxygen saturation above 94%. High flow via a nasal cannula, a face mask, or a non-invasive ventilation (preferably using a continuous positive airway pressure helmet or full-face interface) or bilevel positive airway pressure machine is recommended. Endotracheal intubation should not be delayed if patients develop acute lung injury or ARDS [11]. In the patients who died, the time of admission was not significantly different from those who survived and also required mechanical ventilation. Our study revealed some discrepancies in the criteria for admission to PICUs in Poland depending on the local situation. According to our latest up-to-date admission criteria, admitted patients are usually those diagnosed with a life-threatening condition, resulting mainly from acute respiratory and/or circulatory failure, as well as multiple organ failure syndrome, requiring the use of monitoring and treatment techniques available only in PICUs [12]. Unfortunately, we still admit “too sick” patients to the ICU, despite available guidelines and recommendations [12,13,14]. The COVID-19 pandemic is representative of a particularly difficult time, and the use of the so-called triage is associated with high levels of stress in regard to making a responsible decision [15]. The median age of our patients was 2 years. In the literature, a severe course of illness is most often described in two age groups—in children < 2 years of age [3,16,17] and in adolescents (median 9 years of age) [7,11]. Two multicentre studies in Europe showed that a higher prevalence of neonates required ICU admission than older patients [18,19]. In our analysis, the interquartile range is extensive. Perhaps our group is younger due to the fact that in Poland, all disabled and handicapped children are often hospitalised in the first years of their lives.

Factors correlating with patient prognosis included statistically significant lower oxygen pressure at the time of admission to the ICU, which resulted in lower saturation values, the need for higher oxygen concentrations, a lower oxygen index, and a higher rate of lung damage. Oxygenation index (OI) is used to assess the respiratory capacity of the human body. OI < 200 mmHg in the presence of bilateral parenchymal changes in the lungs corresponding to non-cardiogenic pulmonary edema and the absence of symptoms of increased pressure in the left atrium meet the criteria for ARDS and represent poor prognosis factors.

Graft et al. reported on which children are at the most significant risk for severe complications from COVID-19 infection. Among the comorbid conditions, diabetes and obesity were predictors of severe COVID-19 in children. Of the 66 symptomatic patients, 55% required respiratory support, and 17% required critical care [3]. The severe course of COVID-19 may manifest itself in the form of shock, encephalopathy, myocardial damage, or renal failure, which may be caused by severe coagulopathies and/or a ‘cytokine storm’ [7,10]. Individual components evaluating multi-organ function in our study did not differ between the compared groups, although the total values according to the pSOFA scale were statistically higher in the group of children who did not survive. The SOFA score at admission is useful for predicting outcomes in the PICUs and is more accurate than SIRS for definition of paediatric sepsis. Individual components evaluating multi-organ function in our study did not differ between the compared groups, although according to the pSOFA scale, the total values were statistically higher in the group of children who did not survive. Note the higher markers for inflammation, tissue damage, and microcirculatory failure in non-survivors. Lactates, ferritin, LDH, and troponin were higher in this group of patients.

Circulatory failure and the need for vasoactive drugs in the study population were observed in almost 65% of patients, although in the available literature, myocardial damage and the need for vasoactive drugs were described more often in children with PIMS than with COVID-19 [3,20,21]. Similarly, higher values of inflammatory markers such as CRP and procalcitonin were found more often in the group of children with PIMS than in COVID-19-positive patients [9,22]. Procalcitonin is a prohormone, a precursor of calcitonin, a hormone that plays a major role in calcium homeostasis. Elevated procalcitonin levels may be seen in sepsis and are particularly associated with septic shock and organ dysfunction, thus requiring intervention. A majority of COVID-19 patients have procalcitonin levels in the normal range [23]. Comparing all patients in terms of the criterion of survival, we did not observe a difference; however, the group of patients who survived and were invasively ventilated tended to have increased procalcitonin [23].

Another parameter, leukocytosis, showed normal values in most children with COVID-19 [24], and it was elevated in only about 10% of patients [25]. NT-proBNP is a hormone released by the muscle cells of the heart ventricles when their tension increases, demonstrating increasing loads [26]. In ventilated patients who survived, it was slightly higher on day 1 than in patients who died, but we did not observe this trend for troponin. We observed a trend of a much higher value in all patients who died. It seems to be a marker of myocardial damage and much more sensitive than NT-proBNP [26]. IL-6 is one of the most prominent pro-inflammatory cytokines. Increased levels are recorded in COVID-19 patients, especially those with severe-to-critical disease. Evidence is accumulating for the relevance of IL-6 as a prognostic marker in COVID-19 [26]. Interleukin-6 is one of the most important signalling molecules produced by cells of the immune system. It has been shown that IL-6 is a cytokine with dual activity, which means that on the one hand, it can have an anti-inflammatory effect by activating the classical signalling pathways. On the other hand, it induces a pro-inflammatory effect by activating the so-called trans-signalling pathways, thus activating the immune system. Perhaps for this reason, our results show a different trend—patients who survived had lower levels of IL-6, but those who died in the ventilated group had much higher levels of this marker.

Aspartate aminotransferase was a laboratory marker significantly higher in the group of patients who died. Several studies have reported elevated AST, ALT, and LDH levels [27,28] in children diagnosed with COVID-19 and treated in Paediatric Infectious Diseases Wards. Mania et al. suggested a poor correlation between the level of LDH (lactate dehydrogenase) and CK (creatine kinase) in children requiring intensive care, in comparison to children experience a mild-to-moderate course of COVID-19 [27]. The pathomechanism of liver damage in COVID-19 cases is complex and caused by many factors, such as a hyperactive immune response and “cytokine storm”, systemic inflammatory reaction, hypoxia, hypovolemia, acute respiratory failure, and septic shock [29,30]. Disturbances in the intestinal microbiota (intestinal endotoxemia) are also considered. Drugs such as antivirals, antibiotics, and paracetamol (used in high doses in these patients) may also be potentially hepatotoxic in the course of COVID-19. There are also hypotheses that state that liver damage in the course of SARS-CoV-2 results from the expression of the ACE2 receptor in cholangiocytes [31]. AST levels can also rise in myocardial infarction and muscle damage, usually much more than ALT. Kidney damage can also cause an increase in AST. In the group of children who died, elevated AST levels on day 1 correlated with the occurrence of statistically significant hypoxemia on admission, which could significantly contribute to myocardial and skeletal muscle damage and renal hypoxia.

In children treated for COVID-19 in PICU, it has not yet been possible to determine clear prognostic laboratory exponents, which may result from the limited number of studies and publications. Most likely, the correlation of elevated AST values in patients who died may be a predictor of liver damage coexisting with COVID-19, requiring further research [32]. A more severe course of COVID-19 is more common in children with comorbidities and a positive genetic history [33,34]. The meta-analysis and systemic review based on 27 studies showed that obesity, diabetes, heart disease, chronic lung disease, seizures, and immunodeficiency are classified as risk factors for a severe course of COVID-19 infection [16] The study group was dominated by underweight rather than obese children, which was most likely due to the child’s poor condition and/or cachexia. Obesity is a risk factor for a severe course of COVID-19. One of the probable causes is the chronic inflammation associated with obesity, which interferes with the proper immune response and also affects the blood coagulation process. In addition, obesity is associated with functional impairment of the respiratory system.

In our study group, only 5 children had no comorbidities, and all of them survived. The others had comorbidities; the mortality rate in this group was as much as 40% and was assessed as higher than in the reports of other researchers [6,9]. On the other hand, the mortality rate of children in the PICU compared to all children treated in hospitals due to COVID-19 in this period was 0.1% (28 deaths per 91,000 hospitalised patients), and it does not differ from the mortality rate of children due to the SARS-CoV-2 virus in the US [35]. Although we did not find a statistically significant difference in treatment, steroid and anticoagulant therapies were more frequently used in surviving children, which may have contributed to treatment outcomes. In the initial period of the pandemic, we did not have clear recommendations as to which therapy should be used. Today, recommendations for the use of steroid therapy and anticoagulant treatment in patients with severe COVID-19 are known.

The limitation of our study is the fact that our study group was strictly selected and consisted entirely of patients diagnosed with a very severe course of SARS-CoV-2 infection requiring intensive care, presenting the actual condition of children critically ill due to SARS-CoV-2 infection. Further analysis of this issue is necessary.

## 5. Conclusions

As with adults, children with comorbidities are most frequently at risk of severe SARS-CoV-2 infection. Increasing symptoms of respiratory failure, the need for mechanical ventilation, and persistently high values of aspartate aminotransferase are indicators of poor prognosis.

## Figures and Tables

**Table 1:** Number of patients from hospital participating in the study.

Hospital Name, City	Number of Patients
Upper Silesian Child Health Centre, Katowice	9
University Child Hospital, Lublin	9
University Hospital, Poznan	7
University Hospital Nr 1, Szczecin	4
University Child Hospital, Białystok	4
University Hospital, Rzeszów	3
Copernicus Hospital, Gdansk	2
University Clinical Centre of the Medical University of Warsaw	2
University Children’s Hospital of Cracow	2
University Clinical Hospital in Wroclaw	2
Children’s Memorial Health Institute, Warsaw	1

**Table 2:** Characteristics of the study groups (survived vs. died).

	Survived (*n* = 27)	Died (*n* = 18)	*p*
Sex Females Males	*n* = 9 *n* = 18	*n* = 11 *n* = 7	0.125
Age [months] mean ± SD, median	73.15 ± 75.93, 30.5	71.82 ± 75.18, 26	0.654
BMI Underweight, *n*, % Normal, *n*, % Obese, *n*, %	13 (50.00%) 12 (46.15%) 1 (3.85%)	7 (38.89%) 6 (33.34%) 5 (27.77%)	0.748
Time from positive test to hospitalisation in the ICU [days] mean ± SD, median	2.24 ± 3.73, 1	5.06 ± 10.65, 1	0.608
Comorbidities 1. Neurological disorders, *n*, % 2. Immobilisation, *n*, % 3. Immunosuppressive drugs, *n*, % 4. Congenital heart diseases, *n*, % 5. Respiratory failure, *n*, % 6. None, *n*, %	15 (55.55%) 10 (37.03%) 2 (7.4%) 3 (11.11%) 5 (18.5%) 5 (18.5%)	5 (27.77%) 3 (16.66%) 2 (11.11%) 6 (33.33%) 7 (38.88%) -	0.227
From where the patient was admitted COVID-19 ward, *n*, % Emergency Room, *n*, % Home, *n*, % Paediatric Ward, *n*, % Other ICU, *n*, % Operating Room, *n*, %	6 (22.22%) 5 (18.53%) 5 (18.52%) 9 (33.33%) 1 (3.70%) 1 (3.70%)	6 (33.33%) 2 (11.11%) 4 (22.22) 3 (16.67%) 2 (11.11%) 1 (5.56%)	0.691 *
Oxygen therapy, *n*, %	7 (25.92%)	18 (100%)	0.018 *
Mechanical ventilation, *n*, %	20 (7.07%)	18 (100%)	
Circulatory failure	15 (57.69%)	12 (66.67%)	0.54
Hospitalisation time [days] mean ± SD, median	16.56 ± 13.60, 12	10.82 ± 7.02, 11	0.330
Mortality, *n*, % study group		18 (40%)	

Legend: * statistical significance in Mann–Whitney U test.

**Table 3:** Selected laboratory and respiratory parameters in study groups (survived vs. died).

Parameter (Mean ± SD; Median)	Survived	Died	P in Mann– Whitney U Test
First pH measurement	7.33 ± 0.13, 7.36	7.34 ± 0.09, 7.31	0.763
First pO_2_ measurement [mmHg]	103.84 ± 53.61, 84.4	72.75 ± 49.49, 54.0	0.018 *
First pCO_2_ measurement [mmHg]	52.91 ± 24.49, 47.0	51.68 ± 21.85, 45.5	0.990
First arterial oxygen saturation measurement	92.39 ± 7.91, 95.0	78.43 ± 20.92, 84.0	0.018 *
First Fi0_2_ measurement	0.65 ± 0.30, 0.5	0.89 ± 0.19, 1.0	0.020 *
First-day oxygen index [mmHg]	207.73 ± 137.32, 172.5	121.82 ± 147.37, 57.6	0.003 *
Third-day oxygen index [mmHg]	232.29 ± 179.71, 180.0	145.07 ± 142.96, 96.0	0.006 *
Fifth-day oxygen index [mmHg]	229.86 ± 178.88, 158.5	143.87 ± 135.10, 84.5	0.124
First-day lung injury score	2.52 ± 1.81, 2.0	2.73 ± 1.35, 3.0	0.047 *
Third-day lung injury score	2.62 ± 1.44, 2.3	2.47 ± 1.03, 3.0	0.939
Fifth-day lung injury score	2.23 ± 1.29, 2.15	2.59 ± 0.87, 3.0	0.873
First-day pSOFA	6.22 ± 2.88, 5.5	9.38 ± 3.12, 10.0	0.012 *
Third-day pSOFA	6.19 ± 3.41, 5.0	10.50 ± 3.10, 10.5	0.007^*^
Fifth-day pSOFA	5.78 ± 3.15, 5.0	11.50 ± 4.09, 12.0	0.001 *
WBC ** [×10^9^ per L]	10.86 ± 7.17, 9.69	10.81 ± 10.10, 6.45	0.614
PLT ** [×10^9^ per L]	202.82 ± 113.68, 197.0	207.64 ± 171.74, 142.0	0.832
HGB ** [g/dL]	10.91 ± 3.75, 9.8	10.94 ± 1.51, 10.5	0.178
Procalcitonin ** [ng/mL]	7.21 ± 8.96, 2.34	6.64 ± 17.33, 0.37	0.118
C-reactive protein ** [mg/L]	58.85 ± 106.87, 12.48	57.75 ± 77.61, 17.4	0.365
D-dimer ** [ng FEU/mL]	3573.71 ± 4953.15, 1900	2813.75 ± 3343.10, 1485.5	0.561
Creatynine ** [mg/dL]	0.59 ± 0.87,0.26	0.47 ± 0.32, 0.37	0.456
Urea ** [mg/dL]	48.70 ± 54.35, 32.0	27.23 ± 18.06, 24.0	0.217
Ferritine [µg/L]	2307.18 ± 6288.94, 193.0	1112.53 ± 1431.25, 430.75	0.433
LDH [U/L]	687.78 ± 575.99, 434.5	817.23 ± 678.26,505.0	0.232
Troponin [pg/mL]	49.90 ± 62.55, 24.6	950.94 ± 2293.97, 204.0	0.089
IL-6 [pg/mL]	74.22 ± 149.74,18.085	95.12 ± 124.83, 51.27	0.798
Lactate ** [mmol/L]	3.03 ± 4.23, 1.1	17.76 ± 48.76, 3.26	0.070
AST ** [IU/L]	83.04 ± 132.24, 41.0	112.89 ± 139.33, 66.5	0.028 *
ALT ** [IU/L]	75.87 ± 176.89; 25.0	118.22 ± 281.05, 38.5	0.351

* statistical significance; ** PICU admission day.

**Table 4:** Applied treatment in study groups (survived vs. died).

Number (%)	Survived (*n* = 27)	Died (*n* = 18)	*p* in Chi-Square Test
COVID-19 convalescent plasma	7 (26.92%)	4 (23.53%)	0.803
Tocilizumab	2 (8%)	1 (5.88%)	0.793
Remdesivir	5 (19.23%)	6 (33.33%)	0.288
Steroid therapy	27 (100%)	16 (88.88%)	0.076
Anticoagulants	24 (92.30%)	13 (72.22%)	0.073
Immunoglobulins	7 (25.92%)	8 (44.44%)	0.196
Muscle relaxants	11 (40.74%)	10 (55.55%)	0.329

**Table 5:** Characteristics of the ventilated patients (survived vs. died).

Parameter [Mean ± SD; Median]	Ventilated Patients Who Survived (*n* = 21)	Ventilated Patients Who Died (*n* = 17)	*p* Value
Time between positive test and hospitalisation in the ICU [days]	1.63 ± 2.63, 2.13	5.24 ± 10.59, 1	0.247
First-day oxygen index [mmHg]	206.72 ± 147.89, 153.0	122.70 ± 151.86, 56.0	0.007 *
Third-day oxygen index [mmHg]	232.29 ± 179.71, 180.0	145.07 ± 142.96, 96.0	0.086
Fifth-day oxygen index [mmHg]	229.86 ± 178.88, 158.5	143.87 ± 135.10, 84.5	0.124
First-day lung injury score	2.52 ± 1.81, 2.0	3.0 ± 1.35, 3.0	0.106
Third-day lung injury score	2.62 ± 1.44, 2.3	2.47 ± 1.03, 3.0	0.909
Fifth-day lung injury score	2.213 ± 1.34,2.1	2.59 ± 0.87, 3.0	0.48
First-day pSOFA	6.62 ± 2.78, 6.0	9.17 ± 3.16, 9.0	0.043 *
Third-day pSOFA	6.47 ± 3.33, 5.0	10.33 ± 3.24, 10.0	0.020 *
Fifth-day pSOFA	6.0 ± 3.10, 5.0	11.22 ± 4.24, 11.0	0.005 *
WBC ** [×10^9^ per L]	10.86 ± 7.17; 9.69	18.22 ± 10.10, 6.45	0.868
PLT ** [×10^9^ per L]	202.82 ± 113.68, 197.0	162.0 ± 171.74, 142.0	0.985
HGB ** [g/dL]	10.91 ± 3.75, 9.8	10.2 ± 1.51, 10.5	0.126
Procalcitonin ** [ng/mL]	7.21 ± 8.96, 2.34	0.6 ± 17.33, 0.37	0.118
C-reactive protein ** [mg/L]	58.85 ± 106.87, 12.48	13.73 ± 77.61, 17.4	0.235
D-dimer ** [ng FEU/mL]	3573.71 ± 4953.15, 1900.0	429.0 ± 3343.09, 1485.5	0.897
Creatynine ** [mg/dL]	0.59 ± 0.87, 0.26	0.27 ± 0.32, 0.37	0.231
Urea ** [mg/dL]	48.70 ± 54.35, 32.0	8.0 ± 18.06, 24.0	0.362
Ferritine [µg/L]	1837.64 ± 5594.13, 152.65	1112.53 ± 1431.25, 430.75	0.650
LDH [U/L]	652.88 ± 526.68, 489.0	817.23 ± 678.26, 505.0	0.257
Troponin [pg/mL]	41.65 ± 59.86, 16.95	384.0 ± 2293.97, 205	0.130
NT proBNP [pg/mL]	10565.47 ± 15675.56, 2100.0	918.5 ± 441.94,918.5	0.932
IL-6 [pg/mL]	74.22 ± 149.74, 18.08	7.54 ± 124.83, 51.27	0.798
Lactate [mmol/L]	2.95 ± 3.80, 1.7	17.76 ± 48.76, 3.26	0.092
AST on the 1st day [IU/L]	81.90± 135.85, 41.0	117.23 ± 142.36, 77.0	0.039 *
ALT on the 1st day [IU/L]	72.0 ± 166.48, 25.5	39.0 ± 281.05, 38.5	0.217

* statistical significance in Mann–Whitney U test; ** PICU admission day.

## Data Availability

Department of Anesthesiology and Intensive Care, Upper Silesian Child Health Centre.
